# Pictorial Assessment of Health-Related Quality of Life. Development and Pre-Test of the PictoQOL Questionnaire

**DOI:** 10.3390/ijerph19031620

**Published:** 2022-01-31

**Authors:** Patrick Brzoska, Fabian Erdsiek, Tuğba Aksakal, Maria Mader, Sabahat Ölcer, Munzir Idris, Kübra Altinok, Diana Wahidie, Dennis Padberg, Yüce Yilmaz-Aslan

**Affiliations:** 1 Health Services Research, Faculty of Health, School of Medicine, Witten/Herdecke University, 58448 Witten, Germany; Fabian.Erdsiek@uni-wh.de (F.E.); Tugba.Aksakal@uni-wh.de (T.A.); maria.mader@uni-bielefeld.de (M.M.); olcersabahat@gmail.com (S.Ö.); Mohamed.Idris@uni-wh.de (M.I.); Kuebra.Altinok@uni-wh.de (K.A.); Diana.Wahidie@uni-wh.de (D.W.); Dennis.Padberg@uni-wh.de (D.P.); Yuece.Yilmaz-Aslan@uni-wh.de (Y.Y.-A.); 2 Department of Epidemiology & International Public Health, School of Public Health, Bielefeld University, 33501 Bielefeld, Germany; 3 Department of Nursing and Health Services Research, School of Public Health, Bielefeld University, 33501 Bielefeld, Germany

**Keywords:** quality of life, migrants, pictorial, non-verbal, questionnaire, inventory, equivalence, cognitive interviews, think-aloud

## Abstract

The aim of the present study was to develop a pictorial questionnaire for the assessment of health-related quality of life (PictoQOL) and to examine its content validity and usability across three exemplary population groups of different origin residing in Germany (non-migrants, Turkish migrants and Arabic-speaking migrants). A mixed-methods design combining qualitative and quantitative methods was used, comprising 6 focus group discussions with a total of 17 participants, 37 cognitive interviews and a quantitative pretest with 15 individuals. The PictoQOL consists of a pictorial representation of a total of 15 different situations. Using a visual Likert scale, respondents indicate how much each situation applies to them. Some representations proved to be culturally sensitive and were adapted. Respondents found the use of an additional graphic layer in the form of symbols in addition to pictures helpful for interpretation. The PictoQOL is considered to allow a more accessible assessment and better comparability of HRQOL across different population groups regardless of their literacy level. It is therefore considered to be superior to existing instruments for routine use in health research and practice. Future studies need to examine its convergent and factorial validity.

## 1. Introduction

Health-related quality of life (HRQOL) is a frequently studied patient-reported outcome in health research [1]. Examples include the clinical testing of pharmaceuticals, the evaluation of therapies and healthcare concepts as well as benchmarking processes. Identifying disparities in HRQOL between population groups can also help to inform healthcare strategies aiming to reduce deficits in the health system and to ensure patient-centered healthcare [1]. For this purpose, HRQOL needs to be measured with high validity and reliability. 

HRQOL is usually assessed by means of standardized self- or interviewer-administered questionnaires, e.g., from the SF-36, EuroQol or WHOQOL family [2,3]. These questionnaires have been translated and adapted for a large number of populations and, in part, may also differ in their underlying psychometric measurement models [2]. In population-based studies, the use of such questionnaires is particularly challenging when researchers intend to survey migrants. Since migrants may have a limited proficiency in the language of the country they reside in [4], mother-tongue instruments usually need to be used for survey purposes. Although survey instruments frequently applied to assess HRQOL are readily available in different language versions [5], in most cases, they have only been adapted and validated for the languages and populations of the respective countries where migrants originate from [2,3]. Because of differences in the use of the respective language and linguistic changes over time, these instruments usually cannot be administered to migrants without proper re-adaptation and re-validation [6,7]. For example, questionnaires validated for the population in Turkey are often incomprehensible or misleading for Turkish migrants residing in Germany [6,7]. This can greatly limit the validity of studies. For smaller migrant populations such as Kurdish speakers, almost no validated instruments are available. Re-adapting instruments for migrants or revalidating them for previously unavailable languages requires a lot of financial, time and personnel resources and is also associated with methodical challenges [8]. Given the broad cultural and linguistic heterogeneity of the population, this is usually not feasible in population-based research. As a consequence, certain population groups tend to be excluded from such studies [9,10,11,12]. The lack of available instruments thus contributes to migrants often being underrepresented in health research [13,14,15]. In the absence of alternatives, researchers occasionally use non-validated instruments or translate standardized questions “on the fly” during interviews [16]. This can limit the methodological quality of such studies and compromise their validity. If language- and/or culture-specific instruments are used, cross-group comparability may be limited because of poor measurement equivalence. This can be explained by a differential understanding of translated items between population groups [6,7]. 

One strategy to address the aforementioned challenges can be the use of language-independent questionnaires which are based on pictograms. Such instruments can be applied across different population groups with different language skills and require no or only little re-adaptation [17,18,19]. A language-independent instrument could also take into account the fact that some individuals (irrespective of their cultural or linguistic background) have only limited reading and writing skills and are thus unable to complete language-based questionnaires themselves [20,21]. For them, a questionnaire which relies on pictures/pictograms rather than text would have many advantages. 

Currently, questionnaires which are at least to a certain degree based on pictograms or pictures are occasionally used to assess psychological constructs such as anxiety or various developmental disorders in children and adolescents [22,23,24,25,26,27] Their format, graphic design and the degree of pictogram-/picture-based assessment varies greatly between the different inventories and ranges from drawings that visualize the endpoints of Likert scales [26] to the use of picture stories with only little text. An example for the latter is the Dominic-R [22]. The questionnaire was developed for children aged 6 to 11 years and presents respondents with a set of 89 pictures/drawings of different everyday situations of the boy Dominic, which are designed along the DSM-III-R classification system, covering 62 of the 66 criteria of the DSM-III-R. By means of the questionnaire, respondents are asked to choose those pictures which best describe the situation or state they are currently in. The Dominic-R, as well as a computer-assisted version based on DSM-IV (’Dominic Interactive’) [22], allow examining depressive disorders, attention deficit/hyperactivity disorders, anxiety and social behavior disorders. A comparative study in seven countries showed that the Dominic-R allows valid and reliable assessments of these constructs across different cultural contexts as well as comparisons between countries which are equivalent in terms of their measurement [28]. The Picture Anxiety Test (PAT) [29] uses a similar approach in the field of anxiety disorders, presenting a total of 17 items. The Wechsler Nonverbal Scale of Ability [30] conducts an entirely pictorial assessment (in this case, with respect to the cognitive developmental status of children). Relying on an assessment via a mobile application, the QoL-ME has recently been presented for the assessment of the broader concept of quality of life (QOL) in patients with severe mental health conditions. Similar to the aforementioned approaches, the QoL-ME uses a hybrid (text-/picture-based) navigation and assessment [31]; however, it also explores possibilities to assess some dimensions of QOL without verbal cues. 

Most dedicated HRQOL questionnaires, including those used in assessments of children and adolescents, are language-based or use pictures and illustrations only as visual elements [32]. Based on the Dartmouth COOP Functional Assessment Charts [33], the Stark-QoL questionnaire uses a combination of text and graphical elements to assess quality of life in adults based on six items [34]. The ‘Pictorial Thai Quality of Life’ questionnaire (PTQL) [35] employs a similar approach using 25 items. Some preliminary research into the hybrid (text-/picture-based) assessment of HRQOL based on the EQ-5D has been conducted, exploring possibilities to allow a pictorial assessment of the construct for purposes of economic evaluation [36]. 

To the best of our knowledge, however, no instrument is currently available in which the assessment of HRQOL is conducted mainly language-independent. The aim of the present study was to develop such a pictorial questionnaire for the assessment of health-related quality of life (PictoQOL) and to examine its content validity and usability in three selected population groups residing in Germany. These are non-migrants, Turkish migrants as one of the largest population groups with a migration background in Germany [37] and Arabic-speaking migrants as one of the large population groups which recently immigrated to the country [38]. As the conceptual model for the PictoQOL, we used the measurement model of the SF-36 as one of the most established and widely used HRQOL instruments in healthcare research and practice which also has a high validity across different cultures and languages [39,40,41]. The SF-36 consists of 36 items representing 8 dimensions of HRQOL. The underlying assumption and working hypothesis of the study was that all dimensions of the SF-36 measurement model can be represented well by means of pictorial items. Consequently, we considered eight dimensions of HRQOL in the development of the PictoQOL: physical functioning, physical role functioning, emotional role functioning, vitality, emotional/mental wellbeing, social functioning, pain and general health perception. In this article, we present the PictoQOL and describe its development process. 

## 2. Materials and Methods

### 2.1. Study Design and Recruitment of Study Participants

For the development of the PictoQOL, a participative qualitative design consisting of focus group discussions and cognitive interviews was used. All authors are experienced in qualitative interview studies and have had specific professional training. PB is a professor for health services research and an experienced researcher with a doctoral degree in public health. YYA is an experienced researcher with a doctoral degree in public health. SÖ is an experienced researcher with a doctoral degree in sociology. FE, TA, DP, MM and DW are experienced research assistants and doctoral students. KA and MI are trained research assistants with experience in qualitative research.

Interviewees were recruited from the general population based on theoretical sampling aiming to collect qualitative data with the broadest possible range of information [42,43,44]. Aside from having a Turkish, an Arabic or no migration background, respectively, inclusion criteria for both qualitative approaches were an age of 18 years or older and willingness to participate in the study. Migrants are a hard-to-reach population group. Their recruitment for research is therefore associated with different challenges [45]. To address these challenges in the present study, interviewees were recruited through key persons, i.e., individuals who have a special position in the community of the prospective study participants and hence can facilitate recruitment [46]. No direct prior relationships between interviewers and participants were established and no dropouts or refusals were identified. All participants were informed about the aim of the interviews and the overall study and gave informed consent. 

While we had originally planned to conduct 2 focus group discussions with 9 participants each, we changed the design to 6 focus groups with 2–4 participants each in order to allow a more comprehensive discussion process. For the cognitive interviews, we estimated the necessary sample size to be 45 based on previous experiences in the field [6]. We adjusted the size in the course of the research process accordingly as saturation of information could be achieved before completion of all 45 interviews [47], resulting in a total of 37 cognitive interviews. All interviews were conducted by project staff in German, English, Turkish or Arabic language, in some cases also bilingually, in accordance with the preference of the interviewees. 

Following the development phase, a quantitative pretest of the questionnaire was conducted using a convenience sample of 15 individuals aged 18 years or older.

### 2.2. Development of Pictorial Items by Means of Focus Group Discussions 

Pictorial items were designed with the help of 2 × 3 sequential focus group discussions composed of the three aforementioned population groups. This participatory approach aimed to ensure that the target group is involved in the development process from an early stage on to increase usability, comprehensibility and acceptance of the instrument [48]. Based on the SF-36 HRQOL measurement model, different domains of HRQOL were intended to be visualized by several pictorial items. The PictoQOL was designed as a paper-based questionnaire in greyscale in order to ensure the lowest possible threshold for application in clinical practice and to also facilitate potential administration as a postal survey. The number and presentation format of the pictures as well as the layout of the questionnaire, including the response format, were subject to the focus group discussions.

To stimulate discussion and to guide the creative process, the focus group participants were provided with ‘visual cues’ for each of the eight dimensions of the SF-36 measurement model [49,50]. These consisted of images compiled from online graphics databases and, for example, showed different illustrations of wellbeing, pain, sadness and vitality.

During the first three focus group discussions, participants discussed these cues in terms of comprehension, perception of the images, congruence between images and item contents and possible alternative depictions. On the basis of the results, a pool of potential pictorial items was composed, which was further reduced to a draft version of the questionnaire in the second round of the focus group discussions.

All focus groups were carried out in reserved discussion rooms at the university and took between 50 and 80 minutes. Focus group discussions in German or English were conducted by FE, TA, DW and DP. Focus group discussions in Turkish and Arabic were conducted by FE, MI and KA. All discussions were audio-recorded and supplemented by field notes to provide additional context.

In the development of the PictoQOL, a great emphasis was placed on a diversity-sensitive design of the pictorial items which not only considered cultural and religious aspects but also took into account that other characteristics of social diversity such as gender, age and socioeconomic status can be associated with particular needs and expectations [51] which must be taken into account in the development of survey instruments [52,53]. 

### 2.3. Refinement of Items by Means of Cognitive Interviews

The draft of the pictorial items was subsequently further refined by means of cognitive interviews with 37 individuals (comprising 10 non-migrants, 12 Turkish migrants and 15 Arabic-speaking migrants). The Survey Response Process Model [54] was used as the conceptual framework for the interviews. Correspondingly, the cognitive interviews focused on the understanding and interpretation of the items as well as on the information to be retrieved by respondents in order to form a judgment and to reply to the items [55]. Insights into these stages were gained through a think-aloud approach as well as through verbal probing [56,57]. Both approaches of cognitive interviewing were supported by a structured interview topic guide, developed on the basis of results from other studies in the field [7,58,59,60,61,62,63]. It consisted of questions related to the interpretation and comprehensibility of the pictures and response scales as well as to the overall design and presentation of the questionnaire. Cognitive interviews were conducted at the participants’ home, workplace or another setting of their preference and took between 40 and 60 minutes. Interviews in German were conducted by FE, MM and PB. Cognitive interviews in Turkish were conducted by TA, SÖ and YYA. Cognitive interviews in Arabic were conducted by MI.

### 2.4. Pretest of the Questionnaire

Using a convenience sample of 15 individuals, we tested the draft of the final questionnaire for comprehensibility. Respondents were recruited through snowball sampling and were not among the participants who already took part in the focus group discussions and cognitive interviews during the development of the PictoQOL. A preface with information about the study, contact details and text boxes for feedback on each item and a form for sociodemographic information were added. 

### 2.5. Data Analysis

The focus group discussions and cognitive interviews were audio-recorded, transcribed, translated into German and examined by means of qualitative content analysis. In addition, field notes were taken during the focus groups to complement missing information on selected images. In the cognitive interviews, the individual items (pictures) represented the unit of analysis. For this purpose, the responses obtained using the think-aloud approach and verbal probing were examined separately for each item [47]. For the analysis, a code-tree consisting of main and sub-categories was created by FE, TA, YYA and PB, deductively derived from the structured topic guides and inductively supplemented by further relevant categories. Each category was described by definitions and anchor examples. On that basis, the qualitative data were discussed by all authors in order to minimize a potential researcher bias in the evaluation by avoiding the influence of a one-sided subjective view [64]. In the analysis of the qualitative data, sociodemographic information of the interviewees were taken into account as well. The analysis was facilitated by the qualitative data analysis software MAXQDA 12 (VERBI, Berlin, Germany) [65].

## 3. Results

### 3.1. Focus Group Discussions and Cognitive Interviews

A total of 17 individuals, 8 of whom were women, participated in the 6 focus group discussions. Participants were between 23 and 50 years old, comprising non-migrants, Turkish migrants and Arabic-speaking migrants. An overview of their sociodemographic characteristics is provided in Table S1 (see Supplementary Material). 

Following the development of a draft, the items were further refined in cognitive interviews with 37 individuals (see Table S2 in the Supplementary Material for the sociodemographic characteristics of the interviewees). During the process, we identified several key aspects that guided the development of the images and the questionnaire. In the following, we present the summative results of both consecutive approaches to the development of the PictoQOL.

### 3.2. Complexity of Concepts and Understanding of Images

According to the respondents, simpler or narrower concepts, such as subjective health, were easier to understand from and visualize by images than more complex or conditional concepts, e.g., limitations in role functioning due to impaired physical health. Consequently, while participants mostly agreed on ideas of how to depict good and poor subjective health, most did not initially have any idea of how to depict conditional concepts such as differences in role functioning dependent on personal physical health status. Cognitive interviews showed that reducing role functioning to being able to do mundane tasks in the household was seen as an adequate representation. Physical limitations were represented in a similar way to subjective health, including indications of restricted movement as visualized by a plaster cast. To indicate psychological limitations, symbols for sadness/depression were chosen since they were understood by most participants as an indicator for mental health or psychological wellbeing. Because more complex items were harder to understand, these items also required more reiteration to derive a functional version. 

### 3.3. Response Format of the Questionnaire

**Figure 1:**
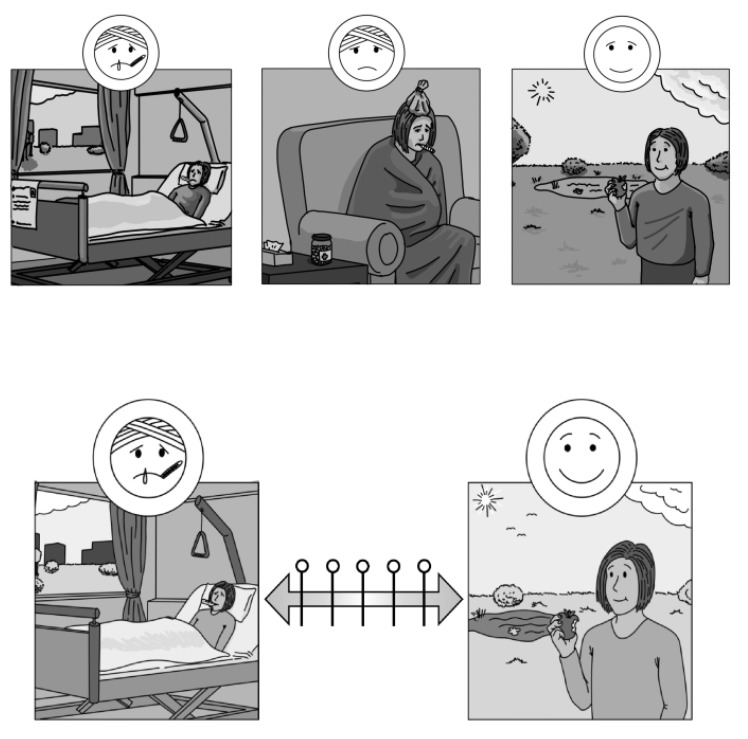
Approaches to the response format of the PictoQOL illustrated using the dimension ‘general health’ as an example (top: original concept visualizing each response category by means of pictures; bottom: final version using a visual 5-point Likert scale response format; Source: own illustration).

We originally planned to design the PictoQOL in such a way that respondents who complete the questionnaire are asked to tick the picture that best describes their condition/situation following the procedure from other pictorial questionnaires such as the Dominic-R [22] or the Picture Anxiety Test (PAT) [29], effectively visualizing each of the SF-36 dimensions with one or more sets of three or four pictures that gradually illustrate changes in the respective dimension (see top part of Fig. 1 for an example based on the dimension ‘general health’). 

**Figure 2:**
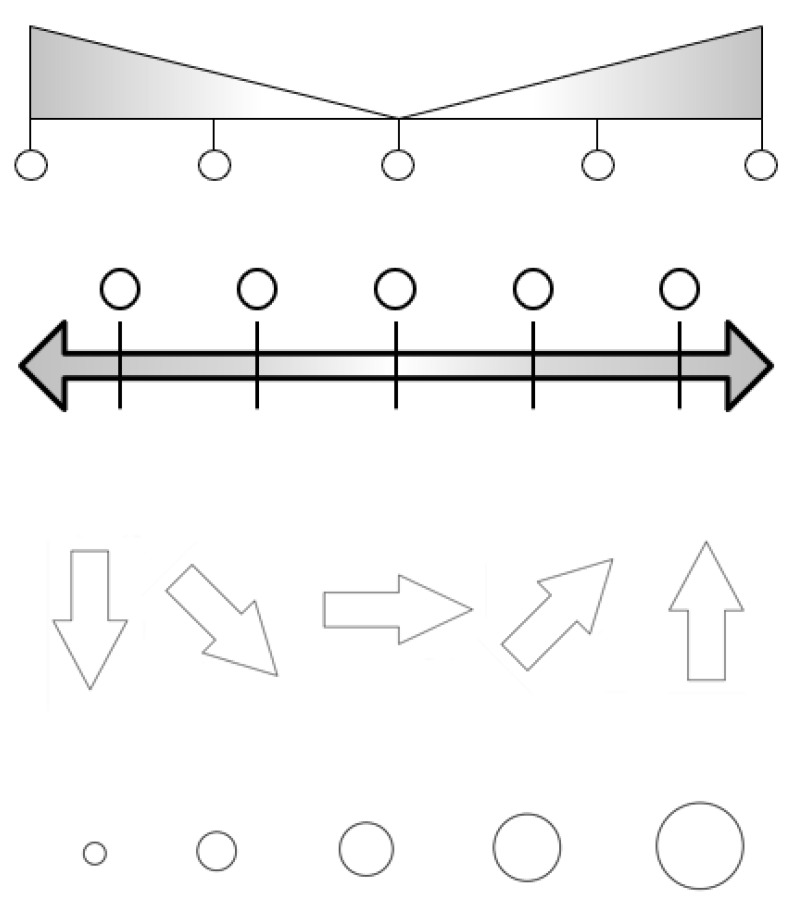
Different types of visual response scales tested during the development of the PictoQOL (Source: own illustration).

In the focus group discussions, it became quickly evident that while the end points for each dimension (e.g., excellent vs. poor health)—with some of the necessary adjustments described above—can be illustrated well using pictures, particularly the middle categories (e.g., very good, good, fair health) are difficult to visualize. This is particularly the case for complex dimensions such as general health, role functioning and social functioning. We therefore dismissed that approach and decided in consultation with the study participants to only illustrate the endpoints with pictures and to apply a graphical 5-point Likert scale in between (see bottom part of Figure 1). Testing different variants of graphical response scales (Figure 2) in focus groups and cognitive interviews, we decided on the use of double-headed arrows with circles representing the different response categories which were favored by study participants. In total, the PictoQOL comprises 15 pictorial items, each consisting of 2 pictures. 

### 3.4. Focal Points and Level of Detail

Participants generally favored a more realistic and detailed depiction instead of very stylized images. The higher level of detail was regarded as key to recognizing pictorial elements and understanding depictions of emotions. These preferences were expressed by all participants. Results from the focus groups showed that to improve recognition of the focal points of the images, the level of detail of different elements (facial expression, gesture/posture, background) needed to be adjusted. While detailed backgrounds were seen as helpful elements to identify social interaction, physical activities and tasks, participants preferred a higher focus on gestures and facial expressions for items addressing emotions and individual reactions to external stimuli. Depictions of sadness, fear, anger and pain were seen as easier to identify when the images focused on the face and body of the depicted person. 

High levels of detail of the background were even regarded as distracting in some cases and negatively affected recognition instead of improving it. In contrast, items focusing more on interaction with physical surroundings or social roles required a detailed background to be recognizable. 

### 3.5. Use of a Recurring Character

The use of a recurring character in the images was considered helpful for identification. Using the same character helped participants to understand that the items represented different questions or inquiries directed at them. When asked about preferences in skin color, gender and age of the persona, participants reported no specific preference. To identify potential differences in perception, we tried out versions with more clearly gendered personas and versions with a mostly androgynous persona. We found that when using an androgynous persona, participants often interpreted these as someone of their own gender, which is why an androgynous character has been used for the final version of the questionnaire.

### 3.6. Culturally Informed Image Contents

Interpretation or preference of visual cues or pictorial elements in some cases differed between participants from different cultural backgrounds. While participants mostly had similar interpretations of the depictions, preferences and perceptions for some elements varied. These differences were mostly related to symbols representing specific concepts or ideas, such as a red cross or a red crescent as illustrations or symbols for healthcare and medicine. In contrast, other symbolic representations were understood by all participants, e.g., the rod of Asclepios as a visual representation of health and healthcare. 

While these differences in perception were not observed for facial expressions, posture and gesture of depicted individuals in some cases led to differences of interpretation. While some participants interpreted stylized lightning bolts as indicators of pain, other participants did not understand these symbols well. Turkish participants also indicated that facial expressions alone were insufficient to depict pain, but instead placing an individual’s hands on the hurting body part was regarded an important indicator of pain.

In addition, the majority of participants generally relied on facial expressions alone to identify specific emotions such as fear or sadness, while Arabic-speaking and Turkish participants put more emphasis on posture. While participants placed different emphasis on the importance of facial expression and posture, preferred postures and facial expressions for specific emotions were similar among the participants. 

In addition, for some depictions, religious beliefs influenced the interpretation of certain aspects. For example, a visual representation of a person sleeping on the side with a relaxed face and their hands under their cheeks was generally seen as a good representation of good sleep quality, while two Muslim participants felt that the person needed to sleep on the right side to represent good sleep and to stay in accordance with the teaching of the Prophet Muhammad.

### 3.7. Use of Additional Symbols

In addition, most participants regarded simplified symbols in addition to the main pictograms helpful. In some cases, these symbols were considered to serve emphasizing the focal point of the respective image, while in other cases the symbols were seen as helpful to identify the differences between the positive and negative endpoints. For example, participants found the use of arrows of different length with equidistant markings to be helpful symbols to represent distance. Participants specifically found symbols helpful when they clearly reflected the opposite end points of the scale. 

### 3.8. Quantitative Pretest

For the pretest, we received responses from 15 participants with different socioeconomic characteristics (see Table S3 in the Supplementary Material). Possible scores of the pretest version ranged from 20 to 100 points. No feedback for the further improvement of the questionnaire has been provided.

## 4. Discussion

HRQOL is an important patient-reported outcome and a relevant determinant frequently studied in health research [1]. Standardized questionnaires readily available for the assessment of HRQOL usually need to be re-adapted prior to their application in migrants [6,7]. In population-based studies, this is often not possible because of resource constraints, and some population groups are excluded from research accordingly. Instead of using language-specific instruments, one strategy to overcome these challenges may be to employ non-verbal questionnaires which are based on pictograms/pictures and which can be employed in different population groups with no or minimal need for adaptation. Such an instrument—the PictoQOL—was developed in the present study.

We designed the PictoQOL with the intention to facilitate an entirely non-verbal assessment of HRQOL. As such, the questionnaire is different from other approaches to pictorial questionnaires developed in the past [31,33,35,36], which often rely on a combination of pictorial items and text. Questionnaires using graphics or photographs with limited text elements are frequently used as self-report pain intensity measures, such as the Faces Pain Scale-Revised (FPS-R), the Oucher photographic and the Wong–Baker FACES Pain Rating Scale (FACES) for children and adolescents [66]. Similar instruments exist to measure social functionality in patients with schizophrenia [67].

Whereas some modules of the QoL-ME, which has been developed for the assessment of QOL in patients with severe mental health conditions [31], are also non-verbal, the authors argue that verbal cues in addition to pictorial items may enhance the clarity of the contents. In our study, after different adjustments were made during the development process, participants in cognitive interviews did not report problems with understanding most of the items, which was further corroborated in the pretest. This overall enhanced clarity may perhaps be explained by the use of repetitive elements and a consistent layout and style, e.g., consisting of additional symbols for each picture as well as a recurring character, and pictures specifically developed for the questionnaire. In addition, participants confirmed the importance of the specific order in which the items were presented. Items were consistently interpreted in reference to previous items and building on information presented in those items. In contrast, the QoL-ME used diverse material selected and composed by respondents from different sources, which differed in style, color and detail between the items. While the advantage of this approach is its highly co-creative character, inconsistencies introduced through the diversity of the material may decrease the clarity of the presentation [68]. 

More complex concepts underlying some dimensions of the PictoQOL, however, still posed some challenges, and we needed to reduce the complexity and scope of some dimensions. This, for example, was the case for role functioning. Future research into the convergent validity of the PictoQOL needs to examine whether these dimensions are still satisfactorily correlated with the respective dimensions of the SF-36.

In the development of the PictoQOL, a great emphasis was placed on the diversity-sensitive designs of all questionnaire elements. It is well known that non-verbal communication may differ between cultures [69] and that also, understanding of non-verbal depictions of different situations as well as the appropriateness of certain symbols is dependent on culture [70]. This also became evident in the focus group discussions with respect to symbols such as a red cross or crescent or the depiction of certain sleep positions. Likewise, preferences of visual representations of abstract concepts were strongly dependent on cultural aspects, confirming the need of a participatory approach to develop the instrument [71]. We examined the contents of all pictures with respect to differences in interpretation between the three groups as well as between men and women and the age range of the respondents and applied several modifications during the development process.

The PictoQOL was designed as a paper-based questionnaire to be completed by the individuals themselves in order to ensure the lowest possible threshold for application in clinical practice. However, a later transfer into an interactive computer-/smartphone-assisted version is possible given the benefits and potential associated with the web- or app-based assessment of self-reported information [72,73]. 

### Strengths and Limitations

Strengths of the study are its participatory approach, the use of different techniques of cognitive interviewing and its focus on three different population groups. However, some limitations need to be considered as well. Although the items performed well in cognitive testing and can be regarded content valid in the three population groups studied, the perspective is still limited. Future research needs to examine whether the items are also comprehensible in other cultural and ethnic groups and also needs to investigate the convergent and factorial validity as well as the measurement equivalence of the PictoQOL. We used a theoretical sampling approach for the recruitment of participants for focus group discussions and cognitive interviews. Still, the maximum age of respondents was only 63 years, as it was particularly difficult to recruit migrants of older age. Future studies must therefore also examine the comprehensibility of the pictorial items in the age groups of individuals 65+. Furthermore, recruitment of individuals for the pretest was only based on a convenience sample and only allows exploratory insights into the performance of the questionnaire which must be complemented by future validation studies based on random samples.

## 5. Conclusions

The PictoQOL has the potential to facilitate an assessment across different population groups irrespective of their linguistic and literacy skills and to allow an anonymous and efficient self-administered assessment of HRQOL also among population groups who usually cannot be surveyed by means of standardized interviews and who therefore are not included in surveys. Future studies need to examine its convergent and factorial validity as well as its measurement equivalence across different population groups. Provided those requirements are met, the PictoQOL would allow valid comparisons of an important quality indicator in healthcare in population groups which currently can hardly be studied by self-administered population-based questionnaires because of limited reading and writing skills [20]. It would thus allow identifying deficits in healthcare for individual population groups and could significantly contribute to ensuring patient-centeredness in healthcare by taking into account the fact that the populations of many countries are becoming increasingly diverse in terms of language and culture [74]. A focus on the majority population is therefore not sufficient and fails to acknowledge the ethical and legal responsibility the health system has toward providing adequate healthcare for the entire population.

## Data Availability

The data presented in this study are available on reasonable request from the corresponding author. The data are not publicly available due to ethical and privacy reasons.

## Supplementary Materials

The following supporting information can be downloaded at: https://www.mdpi.com/article/10.3390/ijerph19031620/s1, Table S1: Socio-demographic characteristics of the interviewees participating in focus group discussions; PictoQOL development study; Table S2: Socio-demographic characteristics of the interviewees participating in cognitive interviews; PictoQOL development study; Table S3: Socio-demographic characteristics of the respondents in the pretest; PictoQOL development study.
