# Supplementary material for: Pictorial Assessment of Health-Related Quality of Life. Development and Pre-Test of the PictoQOL Questionnaire
Source: Int J Environ Res Public Health. 2022 Jan 31;19(3):1620. doi: 10.3390/ijerph19031620 (PMC8835013; doi:10.3390/ijerph19031620)
Supplement: Supplementary file 1 [file ijerph-19-01620-s001.zip › ijerph-1547262-supplementary.pdf]

Supplementary material for "Pictorial assessment of health-related quality of life. Development and pre-test of the PictoQOL questionnaire" (Brzoska et al.)

Table S1. Socio-demographic characteristics of the interviewees participating in focus group discussions; PictoQOL development study.

ID		Age (years)	Sex	Highest educational degree*	Occupation**	Country of birth
**Round 1**						
FG 1	1	29	Female	Bachelor	University student	Syria
	2	No response	Female	No response	No response	No response
	3	No response	Female	No response	No response	No response
FG 2	1	42	Male	Doctoral	Professional	Turkey
	2	24	Female	Upper secondary	University student	Marokko
	3	27	Male	Upper secondary	University student	Germany
FG 3	1	26	Male	Upper secondary	Professional	United Arab Emirates
	2	30	Female	Bachelor	University student	Syria
**Round 2**						
FG 4	1	28	Female	Upper secondary	University student	Syria
	2	24	Female	Upper secondary	University student	Tunesia
	3	23	Male	Lower secondary	Service and sales workers	Jordan
FG 5	1	52	Male	Lower secondary	Craft and related trades workers	Turkey
	2	48	Male	Lower secondary	Associate professionals	Turkey
	3	44	Female	Upper secondary	Housekeeper	Turkey
	4	50	Female	Primary school	Housekeeper	Turkey
FG 6	1	25	Male	Bachelor	University student	Germany
	2	24	Male	Bachelor	University student	Kuwait

* Categorized according to the International standard classification of education 2011 [66] ** Categorized according to the International Standard Classification of Occupations [67].

Table S2. Socio-demographic characteristics of the interviewees participating in cognitive interviews; PictoQOL development study.

ID	Age (years)	Sex	Highest educational degree*	Occupation**	Country of birth
D-01	61	Female	Lower secondary	Service and sales worker	Germany
D-02	27	Male	Lower secondary	Service and sales worker	Germany
D-03	31	Male	Master	Professional	Germany
D-04	42	Female	Upper secondary	Associate professionals	Germany
D-05	63	Female	Lower secondary	None	Germany
D-06	38	Male	Upper secondary	Technicians and associate professional	Germany
D-07	33	Female	Master of Science	Professional	Germany
D-08	25	Male	Upper secondary	Professional	Germany
D-09	54	Female	Upper secondary	Service and sales worker	Germany
D-10	23	Female	Upper secondary	Service and sales worker	Germany
T-01	32	Male	Master	Professional	Turkey
T-02	19	Female	Upper secondary	School student	Germany (Parents: Turkey)
T-03	61	Female	Master	Pensioner	Turkey
T-04	48	Female	Primary	Service and sales worker	Turkey
T-05	61	Male	Bachelor	Skilled agricultural	Turkey
T-06	59	Male	Upper secondary	Craft and related trades worker	Turkey
T-07	61	Female	Lower secondary	Housekeeper	Turkey
T-08	27	Male	Upper secondary	Service and sales worker	Germany (Parents: Turkey)
T-09	38	Male	Upper secondary	Service and sales worker	Germany (Parents: Turkey)
T-10	61	Male	Upper secondary	Craft and related trades worker	Turkey
T-11	62	Female	Primary	Housekeeper	Turkey
T-12	38	Female	Master	Professional	Turkey
S-01	30	Female	Upper secondary	No response	Syria
S-02	28	Male	Upper secondary	Elementary occupation	Syria
S-03	28	Male	Bachelor	University student	Syria
S-04	25	Female	Upper secondary	University student	Syria
S-05	42	Female	Master	Professional	Syria

S-06	27	Male	Bachelor	Service and sales worker	Syria
S-07	35	Female	Bachelor	Service and sales worker	Syria
S-08	30	Male	Primary	Housekeeper	Syria
S-09	63	Female	Bachelor	Pensioner	Syria
S-10	60	Male	*No response*	Pensioner	Syria
S-11	30	Male	Bachelor	University student	Syria
S-12	35	Female	Bachelor	Professional	Syria
S-13	31	Male	Primary	Housekeeper	Syria
S-14	45	Female	Bachelor	Service and sales worker	Syria
S-15	27	Male	Upper secondary	Service and sales worker	Syria

* Categorized according to the International standard classification of education 2011 [66] ** Categorized according to the International Standard Classification of Occupations [67].

Table S3. Socio-demographic characteristics of the respondents in the pretest; PictoQOL development study.

ID	Age (years)	Sex	Highest educational degree*	Occupation**	Country of birth
QT-1	20	Male	Bachelor	University student	Germany
QT-2	27	Male	Upper secondary	No response	Russia
QT-3	45	Female	Bachelor	Professional	USA
QT-4	40	Female	Lower secondary	Service and sales worker	Russia
QT-5	21	Female	Upper secondary	No response	Germany
QT-6	26	Female	Bachelor	University student	Germany
QT-7	25	Male	Lower secondary	Service and sales worker	Turkey
QT-8	22	Male	Upper secondary	Manager	Germany
QT-9	21	Female	Fachoberschulreife	Professional	Germany
QT-10	24	Female	Bachelor	Manager	Afghanistan
QT-11	27	Female	Master	University student/ Professional	Morocco
QT-12	28	Male	Master	Service and sales worker	Morocco
QT-13	51	Male	Master	Professional	Syria
QT-14	46	Female	Master	Professional	Syria
QT-15	31	Male	Bachelor	University student	Syria

* Categorized according to the International Standard Classification of Education 2011 [66] ** Categorized according to the International Standard Classification of Occupations [67].
